# Molecular Imprinting Using a Functional Chain Transfer Agent

**DOI:** 10.3390/molecules27041162

**Published:** 2022-02-09

**Authors:** Phonlakrit Muang-Non, K. Fremielle Lim, Anthony Katselas, Clovia I. Holdsworth

**Affiliations:** 1 Discipline of Chemistry, School of Environmental and Life Sciences, University of Newcastle, Callaghan, NSW 2308, Australia; Phonlakrit.MuangNon@anu.edu.au (P.M.-N.); klim@tpm.com.au (K.F.L.); akat9311@uni.sydney.edu.au (A.K.); 2 Research School of Chemistry, The Australian National University, Canberra, ACT 2601, Australia; 3 The Product Makers, 50/60 Popes Road, Keysborough, VIC 3173, Australia; 4 School of Chemistry, The University of Sydney, Sydney, NSW 2006, Australia

**Keywords:** MIP, molecular imprinting, thioglycolic acid, propranolol, chain transfer

## Abstract

This study demonstrates the feasibility of molecular imprinting using a functional chain transfer agent sans a functional monomer. Ethylene glycol dimethacrylate (EGDMA)-based MIPs were synthesised in the presence of thioglycolic acid (TGA) possessing a carboxylic acid group, capable of interacting with the chosen test template *R*,*S*-(±)-propranolol (PNL) and a labile S-H bond to facilitate an efficient chain transfer reaction. Quantitative ^1^H NMR measurements showed high PNL and TGA incorporation within the MIP, indicating an efficient chain transfer process and a favourable interaction between PNL and TGA. TGA-50, with the lowest amount of CTA, showed the largest imprinting effect and an imprinting factor (IF) of 2.1. The addition of MAA to the formulation improved the binding capacity of PNL to the MIP but also increased NIP binding, resulting in a slightly decreased IF of 1.5. The K_d_ for the high-affinity sites of the TGA/MAA MIP were found to be two times lower (10 ± 1 μM) than that for the high-affinity sites of the TGA-only MIPs, suggesting that the incorporation of the functional monomer MAA increases the affinity towards the PNL template. Selectivity studies, cross-reactivity as well as binary competitive and displacement assays showed the TGA-based MIPs to be highly selective towards PNL against pindolol and slightly competitive against atenolol. The morphologies of the polymers were shown to be affected by the concentration of the TGA, transforming into discrete macrospheres (from small aggregates) at a higher TGA concentration.

## 1. Introduction

The preparation of a molecularly imprinted polymer (MIP) by the self-assembly method is relatively simple, conventionally requiring four essential components: template (i.e., usually the target molecule), functional monomer(s), cross-linker, and porogenic solvent. These formulations as well as their synthetic conditions have been widely studied, tailored, and optimised to enhance binding efficiencies.

For the template to be incorporated into the polymer and form high-fidelity imprints, it must be able to strongly interact with the functional monomer prior to polymerisation in the presence of a high amount of crosslinkers. These interactions can be covalent, non-covalent, electrostatic, or metal ion coordination [1,2,3]. Functional monomers with hydrogen bonding capable groups such as carboxylic acid groups (e.g., methacrylic acid) and heteroaromatic bases (e.g., vinylpyridine) are generally employed in the synthesis [4]. It is also important to control the combining ratio between the functional monomer and template to allow for maximum interaction and provide the most favourable orientation of the functional groups (with respect to the template) for binding to increase MIP selectivity [5,6,7]. In several cases, multiple functional monomers have been used to enhance the interaction with the template [8]. Bespoke stoichiometric functional monomers have also been developed such as the 2,6-bis-(acrylamido)pyridine (BAAPy)-containing amide-based functional groups that allow a 1:1 interaction and an array of hydrogen bonding with an imide-based template such as fluorouracil and barbiturates [9,10,11,12].

The functional monomer provides the functional group required to interact with the template in order to create template-specific cavities. However, a number of studies have shown that the self-assembly MIPs could be synthesised sans functional monomers as long as other components contain functional groups that can interact with the template. Sibrian-Vazquez et al. discovered a simpler approach to MIP formation without the addition of a functional monomer by utilising the functionalised crosslinker N,O-bismethacryloyl ethanolamine (NOBE) [13]. The resulting OMNiMIP was shown to enhance the binding and selectivity properties when compared to the traditional MIP formulated using EGDMA [14]. In another study, a carboxylic acid-functionalised radical initiator, 4,4’-azo-bis-cyanovaleric acid (ACVA) was used to efficiently synthesise MIP without the presence of functional monomers [15]. ACVA MIP was shown to impart superior recognition towards its target *R*,*S*-(±)-propranolol than the MIP prepared using the traditional self-assembly components in the presence of a functional monomer.

In this study, we synthesised and characterised ethylene glycol dimethacrylate (EGDMA)-based MIPs utilising a functional chain-transfer agent (CTA). Thioglycolic acid (TGA), possessing a carboxylic acid group capable of interacting with the chosen test template *R*,*S*-(±)-propranolol (PNL), was used in lieu of a functional monomer. In a chain transfer, the activity of a growing polymer chain is transferred to another molecule [16,17]. Efficient CTAs should have at least one weak bond that is labile to facilitate the occurrence of the chain transfer reaction. Thiols (RSH) such as the TGA utilised in this study are good CTAs because the S-H bond can undergo facile cleavage with high chain transfer rate coefficients, leading to low molecular mass polymers [18,19]. The chain transfer constant (C_x_) values for TGA, representing the efficiency of the chain transfer with respect to propagation (k_tr_/k_p_), was reported to be 0.63–0.75 with methyl methacrylate, the homopolymerisable analogue of EGDMA [20,21].

The mechanism of the chain transfer with crosslinkers such as EGDMA, with two polymerisable vinyl groups in the presence of thiols, is equivalent to that with linear polymers, except that in this instance, it terminates the crosslinking reaction. In the case of TGA, the hydrogen radical cleaved from the S-H bond combines with any radical centre within the EGDMA chain, preventing it from further crosslinking. The other radical moiety from TGA bearing the carboxylic acid group, i.e., •SCH_2_COOH, can react with any free vinyl group within the EGDMA network and reinitiate polymerisation, while at the same time interacting by hydrogen bonding with the template PNL, thus allowing template imprinting to occur within the polymer matrix.

Our results presented here demonstrate the feasibility of molecular imprinting using functional thiol chain transfer agents without any other functional monomer. PNL imprinting was successful, particularly at a low concentration of TGA (IF = 2.1) with high template selectivity against the PNL analogues atenolol and pindolol, according to non-competitive cross-binding as well as competitive binding and displacement tests. Quantitative ^1^H NMR (q-HNMR) measurements also showed high PNL and TGA incorporation within the MIP, indicating an efficient chain transfer process and a favourable interaction between PNL and TGA. Interestingly, the morphologies of the polymers were shown to be affected by the concentration of the TGA transforming into discrete macrospheres (from small aggregates) at a higher TGA concentration.

## 2. Results and Discussion

### 2.1. Polymer Synthesis, Composition, Conversion, and Morphology

*R*,*S*-(±)-PNL-imprinted TGA-MIPs and NIPs were synthesised in the presence of various amounts of TGA: 50 (TGA-50), 100 (TGA-100), 200 (TGA-200), and 400 (TGA-400) μmol per 941 μmol of EGDMA in 3 mL acetonitrile, while keeping the PNL:TGA ratio at 1:4. Polymerisation was conducted at 60 °C for 72 h. After polymerisation, the solid polymers were separated from the acetonitrile solvent by centrifugation. The polymers obtained were easy to process and did not form monoliths that would have required grinding. To extract the PNL template, the MIPs were stirred in 10% acetic acid in methanol, followed by pure methanol. This process was repeated at least three times until no PNL was detected in the methanol wash by ^1^H-NMR based on its aromatic protons at 8.65 ppm.

The polymerisation solution was also collected after polymerisation to determine the residual amounts of PNL, EGDMA, and TGA that had not been polymerised or incorporated into the polymer matrix. Three (3) mL of ACN was added after polymerisation to ensure that a sufficient amount of the post-polymerisation solution could be retrieved for q-HNMR. The dried polymers were subjected to FTIR analysis to estimate the EGDMA double bond conversion.

Table 1 summarises the polymerisation and imprinting results for all TGA MIPs and NIPs. The yields of polymers based on measurements of residual EGDMA by q-HNMR range from 93 to 99%, while as expected, the isolated yields are lower and more variable, ranging from 84 to 97%, which could be attributed to the loss of polymers during the extraction and purification processes.

The degree of double bond conversion ranged from low to moderate, which for a di-polymerisable monomer such as EGDMA, indicates that crosslinking is low and suggests that the resulting polymer is hyperbranched with pendant vinyl groups [22,23]. An increase in the amount of TGA, from TGA-50 to TGA-100, resulted in a 19% increase in the double bond conversion (40–59%), then remained comparable from the TGA-100 to TGA-400 MIPs (56–62%). A higher double bond conversion was observed with higher amounts of TGA, most likely due to a higher number of thiyl radicals formed during the chain transfer that was able to react with the vinyl groups. However, by increasing the amount of TGA, the probability of chain transfer also increases, thus lowering the probability of cross-linking [22] and leading to the lower rigidity of MIPs. The double bond conversion in TGA NIPs did not significantly change (42–46%) and was shown to be lower than that in their respective MIPs, suggesting that the PNL template influenced the double bond conversion in MIPs. The formation of the less flexible PNL:TGA (via the carboxylic acid group of the thiyl radical) cluster possibly enhanced the interaction of the carbon radicals with the available vinyl groups of EGDMA, resulting in a higher double bond conversion.

The incorporation of TGA, measured using the -CH_2_S- ^1^H NMR peak at 3.60 ppm, was high (94–99%) and confirms an efficient chain transfer process and the re-initiation of chain growth by the thiyl radical. While the PNL:TGA feed ratio of 1:4 was preserved at lower amounts of TGA, it was observed to be slightly decreased to 1:5 at higher amounts of TGA, i.e., TGA-200 and TGA-400. This could be due to the increased rate of pre-mature termination when a higher amount of TGA is present, giving shorter polymer chains and making it difficult for PNL to fit within the crosslinked chains. Furthermore, these shorter chains could enhance close-range hydrogen bonding between TGA-derived carboxylic groups, which could prevent the interaction with PNL or introduce steric hindrance between PNL molecules that could otherwise interact with the carboxylic groups, subsequently lowering PNL incorporation.

**Figure 1:**
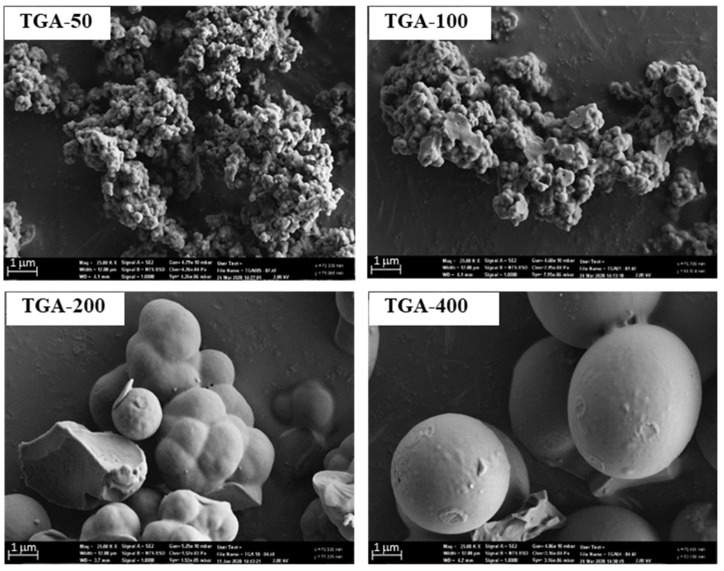
SEM images at 2k magnification of TGA-MIPs produced from different amounts of TGA.

SEM micrographs of vacuum-dried polymers were obtained after template extraction. All TGA NIPs formed small aggregates resembling the morphologies observed for the TGA-50 and TGA-100 MIPs, as shown in Figure 1. However, the TGA-200 and TGA-400 MIPs formed more discrete spherical particles. The morphology of MIPs is largely dependent on the type of synthetic method [24] where spherical particles are obtained when precipitation [25,26] and emulsion [27,28] polymerisation are utilised, but not in bulk imprinting where a limited amount of solvent is used, as in this study. It is noteworthy that the spherical particles were obtained only with the TGA-200 and TGA-400 MIPs but not with their NIP counterparts, suggesting the influence of the PNL template on the morphology, quite possibly by acting as nucleation points [28]. It would seem that the amount of TGA also contributed to the resultant morphology, as the TGA-50 and TGA-100 MIPs with lower TGA content did not form spherical particles. Having higher amounts of TGA could enhance the formation of PNL nucleation points by increasing the extent of chain transfer and chain growth via the carboxylic acid-functionalised thiyl radical where PNL could interact.

### 2.2. Template Binding Studies

#### 2.2.1. Time Binding

Different MIP systems show different rates of analyte reuptake. Hence, prior to undertaking any other rebinding tests, the minimum time required to reach optimal template binding was determined. For this test, 2 mg of the TGA-50 MIP and NIP were incubated in 2 mM of PNL solution at various times: 1, 2, 4, 8, and 16 h. The amount of PNL bound to the polymer was determined based on the difference between the initial and final (after rebinding) concentrations of PNL in solution by q-HNMR.

The results in Figure S1 show plateauing after 2 h, indicating that optimal binding was achieved. For this reason, all subsequent binding studies were run for 2 h. The results also show the MIP binding of PNL to be two times higher than in the NIP (200 μmol/g vs. 98 μmol/g), confirming that the imprinting effect enhances PNL sorption.

#### 2.2.2. Effect of TGA Concentration

**Figure 2:**
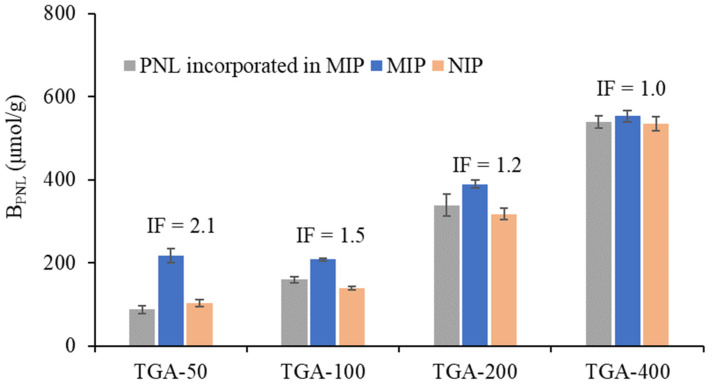
PNL binding of TGA-MIPs and their corresponding NIPs. A total of 2 mg of polymers were incubated for 2 h in 1.0 mL of 2 mM PNL rebinding solution, measured in triplicates for each of the four batches of MIPs synthesised. Post-rebinding solutions were analysed by q-HNMR. IF is the imprinting factor, which is the ratio of PNL bound in MIP to that in NIP. PNL incorporated within the MIP, also measured by q-HNMR (see Table 1), is included for comparison.

The binding performance of the polymers as a function of the TGA concentration was evaluated by incubating 2 mg of the MIPs and NIPs in 2 mM PNL solution for 2 h. The results are shown in Figure 2.

Both TGA MIPs and NIPs exhibited an increase in PNL binding, with an increasing amount of TGA in the feed and incorporated within the polymer. Subsequently, the increase in the number of carboxylic acid attached to the polymer enabled greater interaction and binding with PNL. However, the differences in PNL binding between the MIP and its NIP counterpart became smaller as the amount of TGA increased, giving a decreasing trend for the imprinting factor (IF), from 2.1 (TGA-50) to 1.0 (TGA-400). This indicates that an increase in CTA concentration resulted in minimal up to nil imprinting effect. While the higher concentration of CTA provided more carboxylic acid interaction sites for PNL, the chain transfer decreased chain crosslinking, resulting in the lower rigidity of the polymers. In particular, the TGA-400 MIP showed PNL binding comparable to its NIP, suggesting that only superficial non-selective binding had occurred. The small pore diameter (3.8 Å) obtained for the TGA-400 MIP, even smaller than that of acetonitrile and the PNL molecule, also suggests that high-fidelity PNL imprints were not formed during polymerisation.

Previous studies have shown template rebinding to be significantly lower than template incorporation [9,29,30], which is not the case for the TGA-MIPs. The amount of PNL bound was observed to be higher than the amount of PNL incorporated within the polymer matrix, which may be due to the presence of the oxygen atoms on the EGDMA that could also interact with PNL via hydrogen bonding.

#### 2.2.3. Binding Isotherms: Evaluation of Binding Parameters

The binding characteristics of TGA-50 and TGA-400, identified via their IFs to be the best and poorest performing MIPs, respectively, were further evaluated. Langmuir and Scatchard-binding isotherms (Figure S2) were constructed to obtain the binding parameters given in Table 2. The Scatchard plots for the NIPs only consist of one linear curve, while those for the MIPs show two gradients. These results indicate that the NIPs possess one type of binding sites, whereas the MIPs are more heterogeneous and exhibit two types of binding sites, with the higher affinity sites (steeper slope) most likely introduced by the imprinting process.

As shown in Table 2, the K_d_ associated with the high-affinity binding sites for TGA-50, introduced by the successful imprinting of PNL, is 54 times lower than the K_d_ of its corresponding NIP, which is expected to superficially bind PNL only in the absence of imprints. The MIP also exhibits low affinity binding sites, with a K_d_ comparable to that for the NIP. In the case of the TGA-400 MIP, the K_d_ value of its high-affinity sites is equivalent to that of the high-affinity sites of the TGA-50 MIP; however, the K_d_ value obtained for its low-affinity sites (161 μM) is six times lower than that for the low-affinity sites of the TGA-50 MIP. The K_d_ value of the TGA-400 NIP (75 ± 5 μM) is also 13 times lower than the K_d_ value of the TGA-50 NIP. The favourable low-affinity PNL binding observed in TGA-400 is most likely linked to the higher amount of TGA incorporated in the polymer, resulting in a higher number of carboxylic groups which are held in more flexible highly branched and less crosslinked chains, and are thus able to interact more with PNL. Hence, while TGA-400 was able to imprint and produce selective binding sites of high affinity for PNL, the NIP also possesses superficial binding sites capable of binding PNL favourably and with a capacity equivalent to that of the MIP, resulting in an IF of 1.0 (Figure 2).

The B_max_ for the high-affinity and low-affinity binding sites of the TGA-400 MIP are 5.4 and 1.6 times higher than those of the TGA-50 MIP, respectively. Moreover, the B_max_ for the TGA-400 NIP is 1.6 times higher than that for the TGA-50 NIP. This is expected due to the higher amount of TGA incorporation in TGA-400, allowing for more interactive sites with PNL.

The ΔG_binding_ value represents the thermodynamic relationship between the binding sites and the analyte. ΔG_binding_ for the high affinity sites of the TGA-50 MIP (−27 kJ/mol) and the TGA-400 MIP (−27 kJ/mol) are identical and low negatives, suggesting a more favourable affinity with PNL than with their low-affinity binding sites and those of their counterpart NIPs, which also exhibit comparable ΔG_binding_. It is, however, worth noting that the ΔG_binding_ for the low-affinity sites of TGA-400 are lower than those for the low-affinity sites of TGA-50, suggesting more favourable superficial interaction and binding with PNL at a higher TGA concentration.

### 2.3. Selectivity Studies

**Figure 3:**
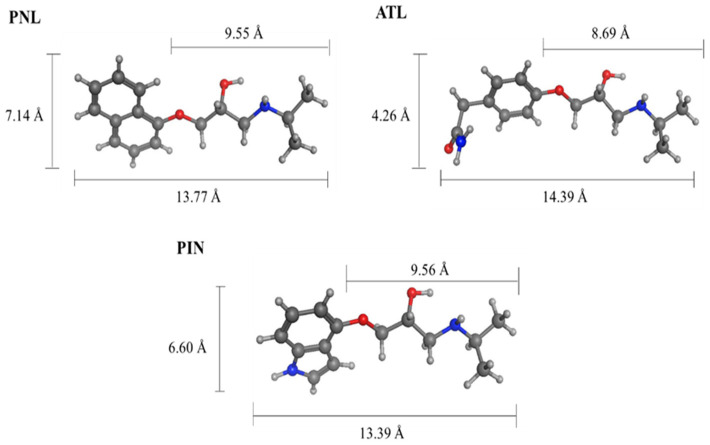
Chemical structures showing the sizes of propranolol (**PNL**), atenolol (**ATL**), and pindolol (**PIN**). Images and sizes generated using the Molecular Operating Environment (MOE) modelling software.

The selectivity of MIPs depends on their ability to maintain the fidelity of the binding sites formed by template imprinting and can be tested by conducting non-competitive and competitive binding assays against template analogue compounds. For this study, non-competitive cross-binding, binary competitive, and displacement binding tests were conducted against atenolol (ATL) and pindolol (PIN) PNL analogues (see Figure 7 for their structures and Figure 3 for their sizes) using the TGA-50 and TGA-400 MIPs.

#### 2.3.1. Non-Competitive Cross-Binding Studies

**Figure 4:**
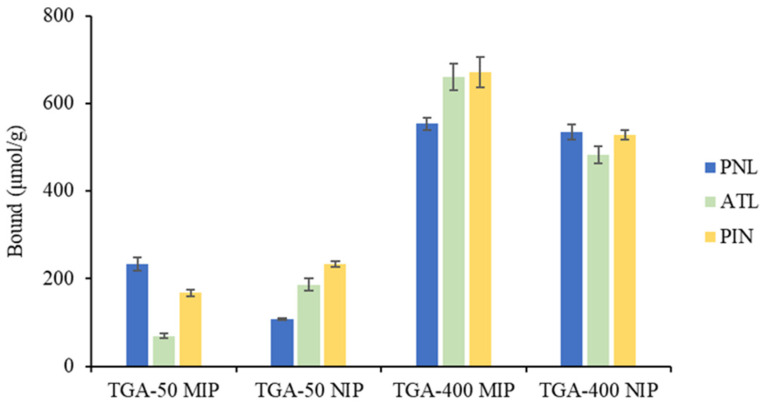
Cross-binding results for the TGA-50 and TGA-400 PNL MIPs and NIPs against atenolol (ATL) and pindolol (PIN). A total of 2 mg of polymer was incubated in 1.0 mL of 2 mM analyte solution for 2 h. All binding tests were conducted in triplicates for each of the four batches of polymers.

The results of the cross-binding tests, where the PNL-imprinted TGA-50 and TGA-400 MIPs and their NIPs were allowed to bind separately with ATL and PIN, are shown in Figure 4. TGA-50 NIP exhibited preferential binding with ATL and PIN, 49% and 42%, respectively, more than PNL. On the other hand, the TGA-50 MIP preferentially bound PNL, 30% and 72% more than ATL and PIN, respectively, confirming the positive effect of imprinting on the selectivity of the PNL template. However, the high amount of PIN that bound to the TGA-50 MIP is indicative of a favourable interaction most likely due to the presence of the >NH in the indole ring that can hydrogen bond with the carboxylic acid groups within the binding sites of the polymer.

The TGA-400 NIP was observed to bind equivalent amounts of PNL, ATL, and PIN, and there was no preferential interaction with any of the compounds, which is characteristic of superficial binding events. The TGA-400 MIP also bound comparative amounts of ATL and PIN, higher than the PNL binding. This is consistent with earlier binding results (Section 2.2.2 and Section 2.2.3), suggesting that high-fidelity PNL-imprinted sites were not formed at a higher TGA feed. The higher uptake of ATL and PIN compared to PNL could be attributed to the presence of >NH in both analogues, which provide additional hydrogen bonding interaction sites that are not present in PNL.

#### 2.3.2. Binary Competitive Binding Studies

**Figure 5:**
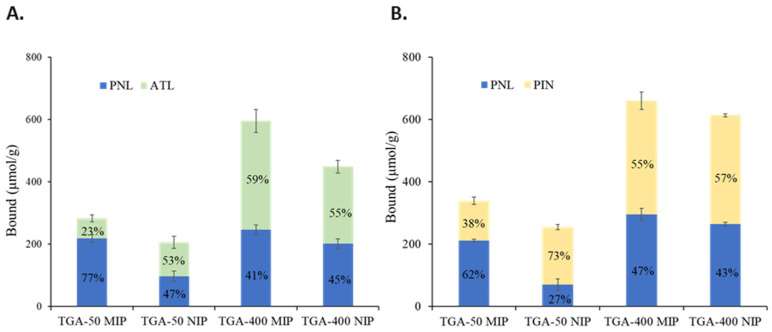
Binary competitive binding results for the PNL binding of the TGA-50 MIP/NIP and the TGA-400 MIP/NIP against ATL and PIN. A total of 2 mg of the polymer was incubated in a 1.0 mL mixture of the 2 mM PNL + 2 mM ATL (**A**) and 2 mM PNL + 2 mM PIN (**B**) solutions for 2 h. All binding tests were conducted in triplicates for each of the four batches of polymers.

The template selectivity of the TGA-50 and TGA-400 MIPs was further assessed by competitive binding assays between the PNL template and an equimolar amount of either the ATL or PIN analogue. The results are shown in Figure 5.

As with the cross-binding results, the TGA-400 MIP and NIP showed no preferential binding towards PNL, ATL, or PIN. On the other hand, while the TGA-50 NIP showed comparable binding between PNL (47%) and ATL (53%) and a slightly higher preference for PIN (73%), the TGA-50 MIP shows a high preference for PNL (77%) over ATL (23%) and a moderate preference for PNL (62%) over PIN (38%). On closer look, the PNL binding observed for TGA-50 in the presence of ATL or PIN is comparable to its binding capacity (217 μmol/g) when PNL is bound on its own (see Figure 2) and suggests that a large extent of the analogue binding occupies superficial binding sites.

#### 2.3.3. Displacement Binding Tests

The strength of the binding interaction between the template and the binding sites in MIPs can be evaluated through competitive displacement binding assays. In this study, the TGA-50 and TGA-400 MIPs were subjected to PNL binding displacement tests against ATL and PIN. The results are shown in Table 3.

In the case of TGA-50, only 26% and 21% of PNL were displaced by ATL and PIN, respectively, while PNL was able to displace 89% and 38% of ATL and PIN, respectively.

For the TGA-50 MIP, the amounts of the displaced analytes (PNL, ATL, or PIN) are quite similar and suggests that there are binding sites that have no preferential analyte binding. The structures of the three analytes in Figure 3 show that half of their structures is identical. It is possible for PNL to be partially imprinted by virtue of its linear ‘tail’ group, resulting in binding cavities that could also fit the linear tail groups of ATL and PIN. These partially imprinted sites could be occupied by PNL, ATL, or PIN in the same manner and could also be displaced by each other.

The TGA-400 MIP, on the other hand, does not exhibit any selectivity behaviour towards the PNL template, consistent with the results from cross-reactivity and competitive binary binding. PNL and its analogues ATL and PIN were shown to be able to displace between 43 and 53% of each other, suggesting that imprinting did not form binding cavities selective towards PNL.

### 2.4. Thioglycolic Acid/Methacrylic Acid-Based MIP (TGA/MAA MIP)

#### 2.4.1. Synthesis, Composition, and Physical Characteristics

To evaluate the effect of the presence of a conventional functional monomer on the binding performance of TGA-based MIPs, methacrylic acid (MAA), a functional monomer capable of interacting with PNL via hydrogen bonding, was incorporated into the polymerisation feed. The reaction feed was analogous to TGA-400 containing 400 μmol of carboxylic acid groups (50 μmol TGA + 350 μmol of MAA) and 2000 μmol of double bonds (2 × 825 μmol EGDMA + 350 μmol of MAA) in 3 mL acetonitrile. The PNL template (100 μmol) was added to the MIP at a 1:4 ratio with respect to the amount of TGA+MAA. The synthesis and extraction processes were as per the TGA MIPs.

High polymer yields, ≥96% from q-HNMR and ≥92% isolated yields, for both MIPs and NIPs were obtained; however, the double bond conversion (~40%) was observed to be lower than that of the TGA-400 MIP but comparable to the TGA-50 MIP. The incorporation of both TGA (97%) and PNL (90%) was high, and the 1:4 PNL:TGA+MAA feed ratio was preserved after polymerisation, resulting in a PNL-to-functional group (i.e., TGA+MAA) interacting ratio of 1:4.

**Figure 6:**
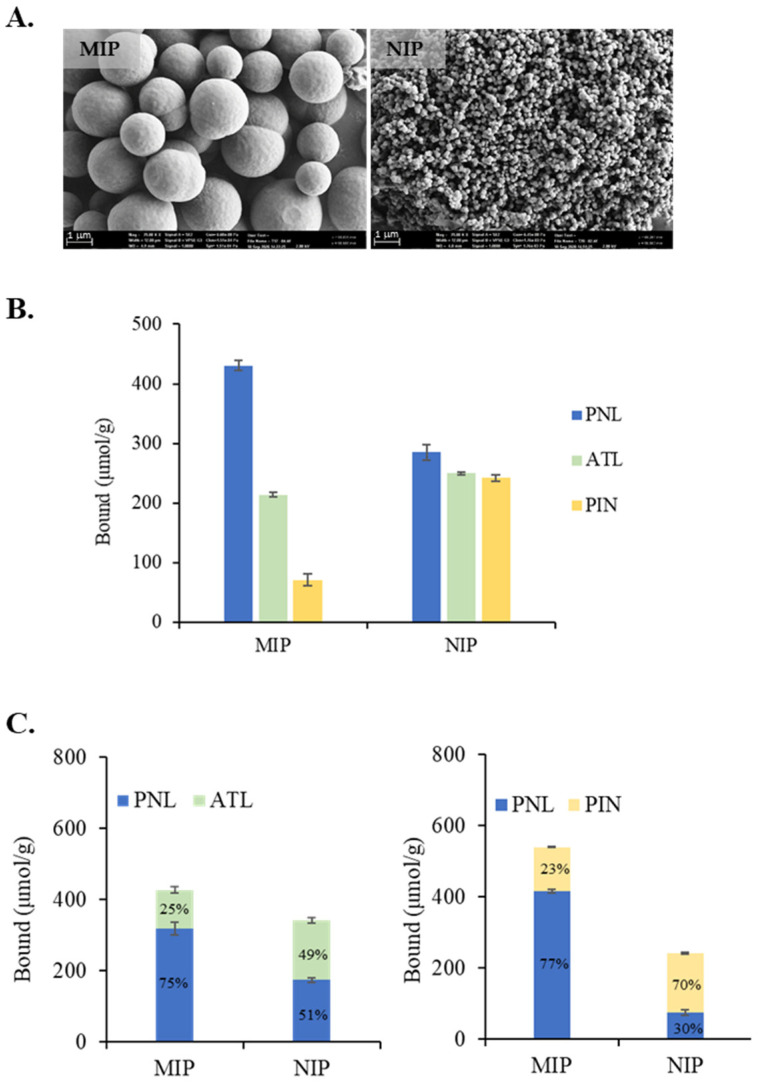
(**A**) SEM images at 25k magnification of the TGA/MAA MIP and NIP; (**B**) Cross-binding results for the TGA/MAA MIP and NIP against atenolol (ATL) and pindolol (PIN); (**C**) Binary competitive binding results for the TGA/MAA MIP and NIP against ATL and PIN. A total of 2 mg of polymer was incubated in 1.0 mL of 2 mM analyte solutions for 2 h.

The SEM images of the TGA/MAA MIP and NIP in Figure 6A show the formation of well-defined spherical particles, with the MIP being 10 times larger than the NIP. A similar morphology was also observed in the TGA-400 MIP, which is most likely due to the ability of the PNL to act as the nucleation point where the crosslinked polymer chains could continuously and evenly grow and form microspheres [28]. The addition of MAA seemed to change the morphology of the NIP, from aggregates to more discrete spherical particles [31,32]. 

#### 2.4.2. Binding Assays

To find the saturation binding of the TGA/MAA system, 2 mg of the MIP and NIP were incubated with a 2 mM PNL solution in acetonitrile, first at 2 h, and then extended to 24 h. The amount of PNL bound to MIP and NIP were evaluated and are shown in Figure S3. The TGA/MAA MIP showed no significant difference in PNL binding after incubating for 2 h and 24 h; however, the TGA/MAA NIP showed a lower amount of PNL binding after 24 h of incubation. The NIP seemed to require more time to equilibrate than its MIP, suggesting that imprinting enhanced the binding equilibration time in MIP. For this reason, all subsequent binding for the TGA/MAA system was carried out for 24 h.

The results presented in Figure S3 show a significant difference in PNL binding between the MIP and NIP at 24 h, with 430 μmol/g and 285 μmol/g PNL bound, respectively, giving an imprinting factor of 1.5 and confirming a positive imprinting effect. Compared to TGA-400, an equivalent MIP where minimal imprinting effect was observed, the imprinting outcome in TGA/MAA was improved with the incorporation of MAA. Additionally, a lower amount of TGA in the system allowed for more crosslinking to occur, imparting higher rigidity to the polymer. The TGA/MAA NIP bound more PNL than the TGA-50 NIP, which was expected as a larger number of carboxylic groups derived from MAA enabled more interaction with PNL. Thus, while both TGA/MAA and TGA-50 used the same amount of TGA, the imprinting factor for TGA/MAA was lower (IF = 1.5) than TGA-50 (IF = 2.1) due to the high PNL uptake by its NIP.

The PNL incorporation of the TGA/MAA MIP is 571 μmol/g, which is comparable to that of the TGA-400 MIP and higher than that of TGA-50, which could be due to the presence of MAA being able to interact with and capture PNL into the polymer network. It is also worth noting that the amount of PNL incorporated into the TGA/MAA MIP is higher than the bound PNL, in contrast to that of TGA-50 and TGA-400. This is reasonable as MAA can also hydrogen bond with neighbouring MAAs and/or TGA carboxylic groups in the polymer, lowering the PNL superficial uptake.

The binding isotherms and Scatchard plots for the TGA/MAA MIP and NIP are presented in Figure S4, and the parameters derived from the plots are given in Table 2. As with the other systems, the Scatchard plot generated for the MIP shows two types of binding sites, with the steeper line (i.e., the one with higher gradient value) representing the high-affinity binding sites, while that for the NIP only shows one type of binding site, as expected. The K_d_ value of the TGA/MAA MIP high-affinity sites is 69 times lower than its low-affinity sites and 45 times lower than its corresponding NIP, illustrating the successful formation of high-fidelity PNL binding sites from the imprinting process. The B_max_ for the TGA/MAA MIP is higher than its NIP, which is expected due to the effect of imprinting, but lower than that for the TGA-400 MIP although higher than that for the TGA-50 MIP. ΔG_binding_ for the high-affinity sites of the TGA/MAA MIP (−28 kJ/mol) is comparable to those for the TGA-50 MIP and the TGA-400 MIP and more favourable than those for its low-affinity binding sites and its NIP.

The non-competitive cross-binding results for the TGA/MAA MIP and NIP against ATL and PIN are shown in Figure 6B. The NIP showed no preferential interaction towards any of the compounds. However, the MIP bound 50% and 83% more PNL than ATL and PIN, respectively, indicating the formation of high-fidelity PNL-selective binding sites by imprinting. ATL is a stronger competitor and bound 66% more than PIN, which could be attributed to the presence of the amide groups allowing more hydrogen bonding between ATL and the polymer binding sites than PIN, and which only contain indole -NH- as an extra hydrogen bonding group.

The selectivity of the MIP in the presence of MAA improved compared to the TGA-400 MIP, with equivalent amounts of functional groups, which was observed to bind non-preferentially to any one of the analytes. The selectivity of the TGA/MAA MIP was most likely enhanced by the lower amount of TGA used, giving a more rigid polymer network that can maintain the specificity of the cavity, and subsequently making it more selective. Additionally, MAA provided a more flexible interaction site for PNL than that of the carboxylic acid group of TGA. It is interesting to note that with the TGA-50 system, PIN was shown to be a stronger competitor than ATL (see Table 3), suggesting that the presence of the indole group that can interact via a π–π interaction, as with the naphthyl ring of PNL, is a predominant interaction in binding.

From the results of the binary competitive binding assays shown in Figure 6C, the TGA/MAA NIP does not show any preference between PNL and ATL, but PIN is preferred over PNL, suggesting the influence of the indole group, capable of a π–π interaction and hydrogen bonding, on PNL binding to the NIP. On the other hand, the TGA/MAA MIP showed high selectivity towards PNL against PIN, but competitive against ATL. In the case of the binding competition between PNL and PIN, PNL bound 416 μmol/g, which is close to the PNL binding capacity of 430 μmol/g (Figure 6B). This suggests that the additional 124 μmol/g PIN binding is mostly superficial, and only 14 μmol/g could have occupied the PNL binding sites. When paired with ATL, PNL binding is only 318 μmol/g and ATL bound an additional 108 μmol/g with their over-all binding equivalent to the observed PNL binding capacity. Thus, ATL is more competitive than PIN, occupying 25% of the binding capacity of PNL as compared to only 3% for PIN.

The results of the competitive displacement binding tests for the TGA/MAA MIP between PNL and its analogues shown in Table 3 are consistent with the selectivity results from the binary competitive tests. ATL and PIN only displaced 26% and 18% of the bound PNL, respectively, whereas PNL was able to displace 59% and 83% of bound ATL and PIN, respectively, from the MIP. These results suggest that ATL is more competitive than PIN, which could be attributed to the presence of the more flexible amide group, allowing for additional hydrogen bonding between ATL and the binding sites within the polymer. While the indole -NH- of PIN is also capable of hydrogen bonding, the ring is not as flexible as the amido group of ATL and is therefore less available.

## 3. Materials and Methods

### 3.1. Materials and Reagents

**Figure 7:**
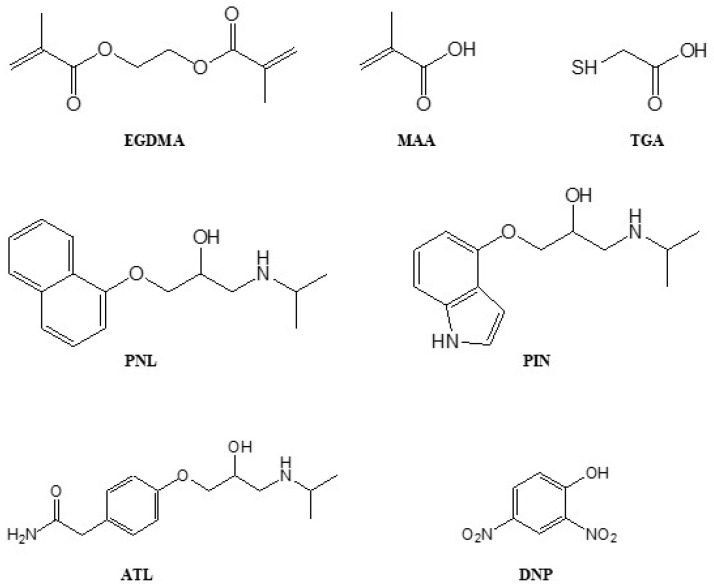
Chemical structures of reagents used for this study.

The 2,2’-azobisisobutyronitrile (AIBN, Dupont, Melbourne, Australia) was recrystallised from methanol before use. Ethylene glycol dimethacrylate (EGDMA, Sigma, Sydney, Australia) and methacrylic acid (MAA, Sigma) were freed from inhibitors using a basic aluminium oxide column. Thioglycolic acid (TGA, Sigma), 2,4-dinitrophenol (DNP, Sigma), atenolol (ATL, Sigma), pindolol (PIN, Sigma), acetic acid (Sigma), methanol (MeOH, Sigma), dichloromethane (DCM, Sigma), acetonitrile (ACN, VWR Chemicals, Radnor, PA, USA), tetrahydrofuran (THF, HPLC grade, Sigma), and deuterated dimethyl sulfoxide (DMSO-d6, Cambridge Isotope Laboratories, Tewksbury, MA, USA) were used as received. Ultrapure nitrogen gas 4.0 was purchased from Coregas (Figure 7).

*R*,*S*-(±)-propranolol HCl (PNL-HCl, Sigma) was converted to the free base *R*,*S*-(±)-propranolol (PNL) by adding 1 g (3.4 mmol) of PNL-HCl into a 10 mL aqueous solution containing 0.6 g (6.8 mmol) NaHCO_3_ (1:2 PNL-HCl:NaHCO_3_ mol ratio) and stirring overnight (24 h). The precipitated PNL was extracted from the aqueous solution with three 10 mL dichloromethane, the solvent evaporated using a rotary evaporator, and the PNL solid dried in vacuo for 24 h.

### 3.2. Synthesis of Polymers

A stock solution containing 28 mL of ACN, 1.76 mL (9333 µmol) of EGDMA, and 93.3 mg (568 µmol) of AIBN was prepared. The solution was placed in a cold sonication bath until all components were completely dissolved. A total of 3.5 mL of the stock solution containing 1167 µmol of EGDMA and 71 µmol of AIBN was transferred to each of the eight 10 mL test tubes, which were then paired.

To each pair of EGDMA solutions, 58 µmol (4.07 µL), 117 µmol (8.14 µL), 233 µmol (16.28 µL), and 467 µmol (32.56 µL) of the TGA chain transfer agent (CTA) were added. Subsequently, PNL (the template) was added to one of the paired test tubes (the MIP) at a 1:4 ratio with respect to the amount of TGA. Thus, 3.8 mg (15 µmol), 7.6 mg (29 µmol), 15.1 mg (58 µmol), and 30.2 mg (117 µmol) of PNL were added, respectively. The remaining 4 tubes with no PNL added were their respective non-imprinted polymers (NIPs).

The solutions were sonicated for 5 min in a cold water bath before 0.5 mL from each test tube was transferred to 5 mm NMR tubes for quantitative NMR (q-HNMR) measurements (i.e., the pre-polymerisation samples). The remaining 3.0 mL solution in all test tubes contained the same amount of EGDMA (941 µmol) and AIBN (57 µmol) and various amounts of PNL (12.5, 25.0, 50.0, and 100.0 µmol) and TGA (50, 100, 200, and 400 µmol) at a 1:4 ratio and labelled as TGA-50, TGA-100, TGA-200, and TGA-400, respectively.

All test tubes were sealed using rubber septa before purging with ultrapure nitrogen gas. To purge, a long purging needle was attached to the nitrogen gas tank and pushed through the rubber septum into the solution mixture. A shorter needle was also pierced through the rubber septum for gas venting. The nitrogen gas was then gently purged into the solution mixture for 10 min. After purging, the test tubes were incubated at 60 °C in a RATEK-HO35 hybridisation oven for 72 h.

After polymerisation, 3 mL of ACN was added to the reaction mixture before sonication for 3 min. A total of 0.5 mL was filtered and then transferred to 5 mm NMR tubes for q-HNMR analysis of the post-polymerisation solutions.

A thioglycolic acid/methacrylic acid (TGA/MAA) mixed system was also prepared following the same procedure, except that 7 mL of ACN, 0.36 mL (1925 µmol) of EGDMA, 23.3 mg (142 µmol) of AIBN, 8.14 µL (117 µmol) of TGA, and 69 µL (817 µmol) of MAA were used as the stock solution. To prepare the MIP, 3.5 mL of the stock solution was transferred to one reaction tube, and 30.2 mg (117 µmol) of PNL was added, ensuring a 1:4 ratio with TGA+MAA (468 µmol). To prepare the NIP, the remaining 3.5 mL of the stock solution was added to a separate reaction tube without adding PNL. As with the other reactions, 0.5 mL of the feed mixture was collected from both reaction tubes for q-HNMR. Both reaction tubes (3 mL) contained the same amount of EGDMA (825 µmol), AIBN (60 µmol), TGA (50 µmol), and MAA (350 µmol). Note that the amount of EGDMA was adjusted accordingly to the amount of MAA in the system, ensuring the same amount of double bonds as the TGA/EGDMA systems.

Four batches of polymers were synthesised for both the TGA/EGDMA and TGA/MAA/EGDMA systems.

### 3.3. Template Extraction

After removing the residual acetonitrile solvent, the MIPs were transferred to 15 mL centrifuge tubes. To extract the PNL template, the polymers were first stirred overnight with 10 mL of the methanol:acetic acid solution (9:1 ratio by volume), followed by 10 mL methanol (MeOH) for 1 h. The process was repeated three times. After the third methanol washing was discarded, 3 mL of MeOH was added to each MIP, and the suspensions were stirred for 30 min before centrifugation for 10 min. A total of 0.5 mL of the methanol supernatant was subjected to ^1^H-NMR to test for the presence of the PNL template. The extraction process was repeated until no PNL peaks (at 8.65 ppm) were observed in the ^1^H-NMR spectrum of the supernatant. After template extraction, the MIPs were dried under vacuum at 40 °C for 48 h. The same washing procedure was also applied to the NIPs.

### 3.4. Determination of Polymer Conversion, Template, and Chain Transfer Agent Incorporation Using Quantitative ^1^H NMR (q-HNMR)

The pre- and post-polymerisation solutions were subjected to q-HNMR to estimate the amount of components incorporated in the polymer by comparing the amounts left in the post-polymerisation solutions to the initial amounts in the pre-polymerisation solutions. Three (3) mL of additional ACN was added after polymerisation to ensure that a sufficient amount of the post-polymerisation solution could be retrieved. A total of 0.5 mL of the reaction solution was placed in the NMR tube, while 10.0 mM of the DNP external standard in DMSO-d6 was placed into a 5 mm probe coaxial insert. The ^1^H-NMR spectra were obtained using Bruker Avance III (Billerica, MA, USA) 400 or 600 MHz-NMR spectrometers. Figure S5 shows examples of the ^1^H-NMR spectra of polymerisation solutions, showing peaks used for quantitative analysis: internal standard (DNP, Ar-H signal) at 8.65 ppm, template (PNL, Ar-H signal) at 8.70 ppm, cross-linker (EGDMA, -CR=CH_2_ signal) at 6.02 ppm, functional monomer (MAA, -CR=CH_2_ signal) at 5.90 ppm, and chain transfer agent (TGA, HS-CH_2_- signal) at 3.60 ppm.

By using a known concentration of DNP (10 mM) as an external standard and a known amount of x component, i.e., template, cross-linker, or chain transfer agent, the response factor (RF) can be calculated using equation (1) [33]:

RF = (C_x_ × I_DNP_ × Hx)/(C_DNP_ × I_x_ × H_DNP_) (1)

where Cx and C_DNP_ are the initial concentrations of the x component and DNP, respectively. I_DNP_ is the integration of the external standard, whereas I_x_ is the integration of component x. H_DNP_ and H_x_ are the number of protons responsible for the integrated peak.

The concentration of component x left after polymerisation, Cx, can then be determined by rearranging Equation (1) with a known RF.

### 3.5. Determination of Double Bond Conversion

The double bond conversion, measured by calculating the difference in the IR absorbance of >C=C< (with respect to -C=O) between the EGDMA monomer and the polymer, can be correlated to the polymer conversion of EGDMA. The ratio of the absorbance of -C=O (1730 cm^−1^) and >C=C< (1650 cm^−1^) in the polymers to that of the pure EGDMA monomer gives the residual double bonds in the polymer and hence the double bond conversion according to Equation (2). IR spectra were generated using Perkin Elmer Spectrum Two FT-IR (MODEL L160000R, Waltham, MA, USA).

(2) $$\mathrm{Double}\;\mathrm{bond}\;\mathrm{conversion}=100\times \left[1-\frac{{\left(\frac{A_{C=C}}{A_{C=O}}\right)}_{\mathrm{polymer}}}{{\left(\frac{A_{C=C}}{A_{C=O}}\right)}_{\mathrm{EGDMA}}}\right]$$

### 3.6. Polymer Morphology

The dried polymers were first coated with platinum using an SPI-module sputter coater three times at a 45-degree angle prior to scanning electron microscopic analysis using Zeiss SEM Gemini (Oberkochen, Germany). The images of the polymers were obtained using 2–100 k magnification at a 2 kV electron beam.

### 3.7. Rebinding Studies

#### 3.7.1. Time Binding

Time binding experiments were conducted using 2.0 mg of TGA-50 MIP and NIP. The polymers were incubated with 1.0 mL of a 2 mM PNL rebinding solution at different times: 1, 2, 4, 8, and 16 h, while agitating in the Intelli-mixer RM-2 rotator. After binding, the samples were centrifuged (3200 rpm) for 10 min, and 0.5 mL of the supernatant was used for q-HNMR to measure the amount of PNL left in the binding solution. A total of 10.0 mM DNP in DMSO-d6 in a 5 mm probe coaxial insert was used as the external standard. Equation (1) was used to calculate the response factor RF and the amount of PNL left in the solution (Cx), respectively. The time binding test was conducted in 4 replicates, i.e., 1 replicate x 4 batches.

#### 3.7.2. Binding Isotherms

A total of 2.0 mg of MIP and NIP were incubated in 1.0 mL at various concentrations, between 0.25 and 4.00 mM, of PNL in acetonitrile for two hours. After 10 min of centrifugation, 0.5 mL of the supernatant was used for q-HNMR, with 10 mM DNP standard in the 5 mm probe coaxial insert as the external standard, to measure the amount of PNL left in the binding solution using equation (1). All binding tests were conducted in 3 replicates per batch of polymer, i.e., a total of 3 × 4 batches = 12 replicates.

The binding isotherms were converted to Scatchard plots to determine the dissociation constants (K_d_) and the maximum number of binding sites (B_max_) for the TGA-50, TGA-400 TGA/MAA systems. By calculating the amount of PNL bound to the polymer (B_PNL_) and the concentration of PNL left in the solution (Free_PNL_) using Equation (1), K_d_ and B_max_ could be determined from Equation (3).

(3) $$\frac{B_{\mathrm{PNL}}\;\left(\mu \mathrm{mol}\cdot g^{-1}\right)}{{\mathrm{Free}}_{\mathrm{PNL}}\;\left(\mu M\right)}=\frac{B_{\max\;}}{K_{d}}-\frac{1}{K_{d}}{\;B}_{\mathrm{PNL}}\;\left(\mu \mathrm{mol}\cdot g^{-1}\right)$$

#### 3.7.3. Cross-Binding Tests

A total of 2.0 mg of MIP and NIP were incubated in 1.0 mL of 2.0 mM atenolol (ATL) or pindolol (PIN) solution in acetonitrile for 2 h. After 10 min of centrifugation, 0.5 mL of the supernatant was used for q-HNMR to measure the remaining ATL or PIN in the solution, with 10 mM of the 2,4-dinitrophenol (DNP) in DMSO-d6 in a 5 mm probe coaxial insert as the external standard. Figure S6 shows the peaks used to quantify ATL and PIN by q-HNMR, applying equation (1): PNL—Ar-H signal at 8.70 ppm, DNP—Ar-H signal at 8.60 ppm, ATL—Ar-H signal at 7.60 ppm, and PIN—Ar-H signal at 6.95 ppm. All binding tests were conducted in 3 replicates per batch of polymer, i.e., a total of 3 × 4 batches = 12 replicates.

#### 3.7.4. Competitive Binary Binding Tests

A total of 2.0 mg of the MIPs and NIPs of TGA-50, TGA-400, and TGA/MAA were incubated in 1.0 mL of a mixture of 2 mM PNL and 2 mM ATL in acetonitrile and were subsequently shaken for 2 h. After centrifugation, the PNL and ATL remaining in solution were measured by q-HNMR using 0.5 mL of supernatant from peaks identified in Figure S6. The test was repeated for the PNL/PIN mixture. All binding tests were conducted in 3 replicates per batch of polymer, i.e., a total of 3 × 4 batches = 12 replicates.

#### 3.7.5. Displacement Binding Tests

A total of 2.0 mg of the TGA-50, TGA-400, and TGA/MAA MIPs were incubated with 1.0 mL of 2 mM ATL solution in acetonitrile and were shaken for 2 h. The supernatants were gently decanted after centrifugation (10 min) to avoid polymer loss. A total of 0.5 mL of the supernatants were kept for q-HNMR, and the rest were discarded. The polymers were then further incubated with 1.0 mL of 2 mM PNL solution in acetonitrile for another 2 h. The test samples were again centrifuged for 10 min, and 0.5 mL of the secondary supernatants were collected. Both supernatants containing ATL (from 1st incubation) and PNL (from 2nd incubation) were analysed by q-HNMR and quantified using Equation (1). The displacement binding process was repeated for another 2.0 mg of the TGA-50, TGA-400, and TGA/MAA MIPs, except that the first incubation was with 1.0 mL of 2 mM PNL solution followed by 2 mM ATL in acetonitrile. Displacement binding tests following the above protocol for TGA-50, TGA-400, and TGA/MAA MIPs were also conducted between PNL and its PIN analogue. All binding tests were conducted in 3 replicates per batch of polymer, i.e., a total of 3 × 4 batches = 12 replicates.

## 4. Conclusions

This study has demonstrated the feasibility of molecular imprinting using a functional thiol chain transfer agent, sans the conventional functional monomer, with EGDMA. All MIPs and NIPs synthesised with TGA as CTA showed high polymer conversion and high TGA incorporation, keeping the PNL:TGA ratio from 1:4 (as with the feed) to 1:5. The extent of the double bond conversion ranged from 40 to 62% and was found to increase with increasing TGA. PNL TGA-MIPs prepared in the presence of MAA as functional monomer, i.e., TGA/MAA, were also obtained in high yields, with the double bond conversion comparable to TGA-50 and preserving the PNL: TGA+MAA (i.e., total functional group) ratio of 1:4.

The morphology of the TGA MIPs, but not the NIPs, was shown to be affected by the concentration of the TGA, changing from small aggregates to discrete macrospheres at a higher TGA concentration, which was attributed to the presence of the template PNL acting as a nucleation point. In the presence of MAA, well-defined spherical particles were formed with the MIP, observed to be 10 times larger than the NIP, again suggesting the influence of PNL on the MIP morphology and particle size, as with the TGA-400 MIP.

While PNL binding was observed to increase with the increasing amount of TGA, TGA-50 with the lowest amount of CTA showed the largest difference in binding between MIP and NIP, resulting in the highest imprinting factor (IF) of 2.1. An increase in the CTA content resulted in less efficient imprinting, which could be attributed to a decrease in crosslinking and an increase in the extent of the branching of the polymer chains, resulting in lower polymer rigidity. While MAA improved the binding of PNL to the TGA-MIP, it also introduced additional superficial PNL binding to the NIP, resulting in a slightly enhanced IF of 1.5, intermediate between TGA-50 and TGA-400. Worthy of note is the fact that the TGA/MAA formulation contained carboxylic acid functional groups (both from TGA and MAA) equivalent to TGA-400 but a lower TGA content equivalent to TGA-50.

The Scatchard plots derived from the binding isotherms confirm the existence of two types of binding sites for the MIPs: the high-affinity sites characterised by steeper gradients, more favourable ΔG_binding_, and lower K_d_s, and the low-affinity sites with gradients, ΔG_binding_, and K_d_s comparable to their corresponding NIPs. The K_d_s for the high-affinity sites of the TGA-50 and TGA-400 MIPs are equivalent (19 ± 2 μM and 21 ± 2 μM, respectively), indicating that molecular imprinting does impart high-affinity binding sites to MIPs, although their selectivities cannot be assessed simply by evaluating their K_d_s. The K_d_ for the high-affinity sites of the TGA/MAA MIP was found to be two times lower (10 ±1 μM) than that for the high-affinity sites of the TGA-only MIPs, suggesting that the incorporation of the functional monomer MAA increases the affinity towards the PNL template.

All selectivity studies: cross-reactivity, binary competitive, and displacement assays, demonstrate that the TGA-50 and TGA/MAA MIPs are highly selective towards PNL than the TGA-400 MIP against PIN and slightly competitive against ATL. The presence of the more flexible amide group, as opposed to the indole -NH- of PIN, allows ATL to be more competitive than PIN due to its capacity for additional hydrogen bonding (three per amide group) with the binding sites within the polymer.

While a number of studies have reported better performing PNL MIPs utilising MAA with TRIM and DVB as crosslinkers [34,35,36], the IF of 2.1 that we have obtained is comparable to reported imprinting efficiencies for MAA/EGDMA-based PNL MIPs [37,38], albeit with differences in MIP synthetic methods.

## Figures and Tables

**Table 1:** Polymerisation and PNL-imprinting results with TGA MIPs and NIPs.

Sample ^1^		Incorporation in Polymer ^2^ % (μmol/g)		PNL:TGA in Polymer	Double Bond Conversion ^3^ (%)
		PNL	TGA		
TGA-50	MIP	96 ± 2 (64 ± 1)	99 ± 1 (268 ± 1)	1:4	40 ± 3
	NIP		99 ± 1 (268 ± 1)	-	44 ± 2
TGA-100	MIP	89 ± 1 (129 ± 7)	96 ± 1 (558 ± 23)	1:4	59 ± 2
	NIP		99 ± 1 (536 ± 1)	-	42 ± 2
TGA-200	MIP	86 ± 2 (240 ± 2)	96 ± 1 (1079 ± 8)	1:5	62 ± 1
	NIP		99 ± 1 (1073 ± 1)	-	48 ± 1
TGA-400	MIP	72 ± 1 (393 ± 6)	94 ± 1 (2039 ± 5)	1:5	56 ± 2
	NIP		98 ± 1 (2100 ± 15)		46 ± 2

^1^ Samples TGA-50, TGA-100, TGA-200, and TGA-400 contain 50, 100, 200, and 400 μmol of TGA in the polymerisation feed, respectively; ^2^ Calculated from q-HNMR in comparison to amounts in polymerisation feed using Equation (1); ^3^ Calculated from FTIR using Equation (2) based on the difference in -C=C- absorbance between the EGDMA monomer and the polymer normalised against the -C=O absorbance.

**Table 2:** Binding and thermodynamic parameters derived from the Scatchard plots for TGA-50, TGA-400, and TGA/MAA MIPs and NIPs.

Sample		K_d_^1^ (μM)		B_max_^2^ (μmol/g)		ΔG_binding_^3^ (kJ/mol)	
		High Affinity	Low Affinity	High Affinity	Low Affinity	High Affinity	Low Affinity
**TGA-50**	MIP	19 ± 2	1217 ± 138	92 ± 8	500 ± 27	−27 ± 3	−17 ± 2
	NIP		1219 ± 81		306 ± 7		−17 ± 1
**TGA-400**	MIP	21 ± 2	161 ± 20	498 ± 50	715 ± 72	−27 ± 3	−22 ± 3
	NIP		75 ± 5		435 ± 23		−23 ± 2
**TGA/MAA**	MIP	10 ± 1	685 ± 24	258 ± 23	505 ± 75	−28 ± 1	−18 ± 3
	NIP		452 ± 36		301 ± 21		−19 ± 2

^1^ K_d_ = −1/K_a_, K_a_ obtained from the gradient of the line in the Scatchard plot; ^2^ Calculated using Equation (3); ^3^ ΔG_binding_ = −RT ln K_d_, T = 298 K, R = 8.314 JK^−1^ mol^−1^.

**Table 3:** Displacement binding tests between template PNL and analogues ATL and PIN to the TGA-50,TGA-400 and TGA/MAA MIPs.

TGA-50 MIP						
	Analyte	Bound (μmol/g)	Displaced μmol/g (%)	Analyte	Bound (μmol/g)	Displaced μmol/g (%)
1st binding	PNL	257 ± 2		PNL	246 ± 6	
2nd binding	ATL	65 ± 2		PIN	116 ± 7	
Displacement	PNL		67 ± 5 (26%)	PNL		51 ± 6 (21%)
1st binding	ATL	56 ± 4		PIN	159 ± 6	
2nd binding	PNL	160 ± 6		PNL	162 ± 3	
Displacement	ATL		50 ± 1 (89%)	PIN		60 ± 2 (38%)
**TGA-400 MIP**						
	**Analyte**	**Bound (μmol/g)**	**Displaced μmol/g (%)**	**Analyte**	**Bound (μmol/g)**	**Displaced μmol/g (%)**
1st binding	PNL	585 ± 6		PNL	581 ± 15	
2nd binding	ATL	579 ± 3		PIN	413 ± 25	
Displacement	PNL		315 ± 11 (54%)	PNL		297 ± 7 (51%)
1st binding	ATL	647 ± 4		PIN	636 ± 19	
2nd binding	PNL	418 ± 17		PNL	400 ± 7	
Displacement	ATL		348 ± 2 (54%)	PIN		289 ± 3 (45%)
**TGA/MAA MIP**						
	**Analyte**	**Bound (μmol/g)**	**Displaced μmol/g (%)**	**Analyte**	**Bound (μmol/g)**	**Displaced μmol/g (%)**
1st binding	PNL	399 ± 13		PNL	407 ± 9	
2nd binding	ATL	171 ± 4		PIN	97 ± 10	
Displacement	PNL		104 ± 3 (26%)	PNL		75 ± 2 (18%)
1st binding	ATL	204 ± 7		PIN	86 ± 5	
2nd binding	PNL	372 ± 20		PNL	415 ± 8	
Displacement	ATL		120 ± 4 (59%)	PIN		71 ± 8 (83%)

Binding assays used 1 mL of 2 mM solutions in acetonitrile. All binding tests were conducted in triplicates for each of the four batches of polymers.

## Data Availability

The data presented in this study are available on request from the corresponding author.

## Supplementary Materials

The following supporting information are available online. Figure S1: Time-binding experiment using the TGA-50 MIP and NIP as test samples. Figure S2: Langmuir binding isotherms (i) and Scatchard plots (ii) for the TGA-50 and TGA-400 MIPs and NIPs. Figure S3: Time-binding tests for the TGA/MAA MIP and NIP. Figure S4: Langmuir binding isotherms (i) and Scatchard plots (ii) for the TGA/MAA MIP and NIP. Figure S5: ^1^H-NMR spectra of the: (a) TGA-400 MIP, (b) ME-400 MIP, and (c) TGA/MAA MIP pre-polymerisation solutions showing peaks used for quantitative analysis. Figure S6: ^1^H-NMR spectra of (a) 2 mM pindolol (PIN), (b) 2 mM atenolol (ATL), and (c) 2 mM propranolol (PNL), with 10 mM 2,4-dinitrophenol (DNP) in DMSO-d6 as external standard in a 5 mm probe coaxial insert.

## Sample Availability

Samples of the MIP materials are available from the authors.
