# Supplementary material for: Molecular Imprinting Using a Functional Chain Transfer Agent
Source: Molecules. 2022 Feb 9;27(4):1162. doi: 10.3390/molecules27041162 (PMC8877225; doi:10.3390/molecules27041162)
Supplement: Supplementary file 1 [file molecules-27-01162-s001.zip › molecules-1574980-supplementary.pdf]

Molecular Imprinting Using a Functional Chain Transfer Agent

Phonlakrit Muang-Non[1,2], K. Fremielle Lim[1,3], Anthony Katselas[1,4] and Clovia I. Holdsworth[1,*]

[1] Discipline of Chemistry, School of Environmental and Life Sciences, University of Newcastle, Callaghan NSW 2308 Australia
[2] Research School of Chemistry, The Australian National University, Canberra ACT 2601 Australia; Phonlakrit.MuangNon@anu.edu.au
[3] The Product Makers, 50/60 Popes Road, Keysborough VIC 3173 Australia; klim@tpm.com.au
[4] School of Chemistry, The University of Sydney NSW 2006 Australia; akat9311@uni.sydney.edu.au
* Correspondence: clovia.holdsworth@newcastle.edu.au; Tel.: +612 4921 5481

Supplementary Data

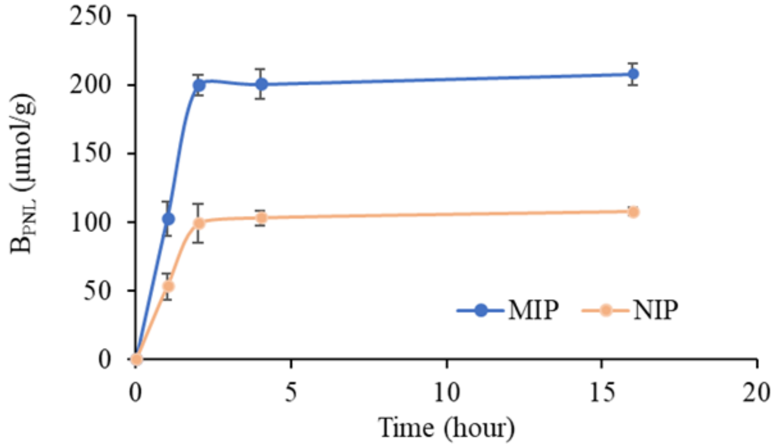

Figure S1. Time-binding experiment using TGA-50 MIP and NIP as test sample. 2.0 mg polymers were incubated at different times with 1.0 mL of 2 mM PNL solution. Rebinding was quantified by q-HNMR. All binding tests were conducted in single run for each of 4 batches of polymers.

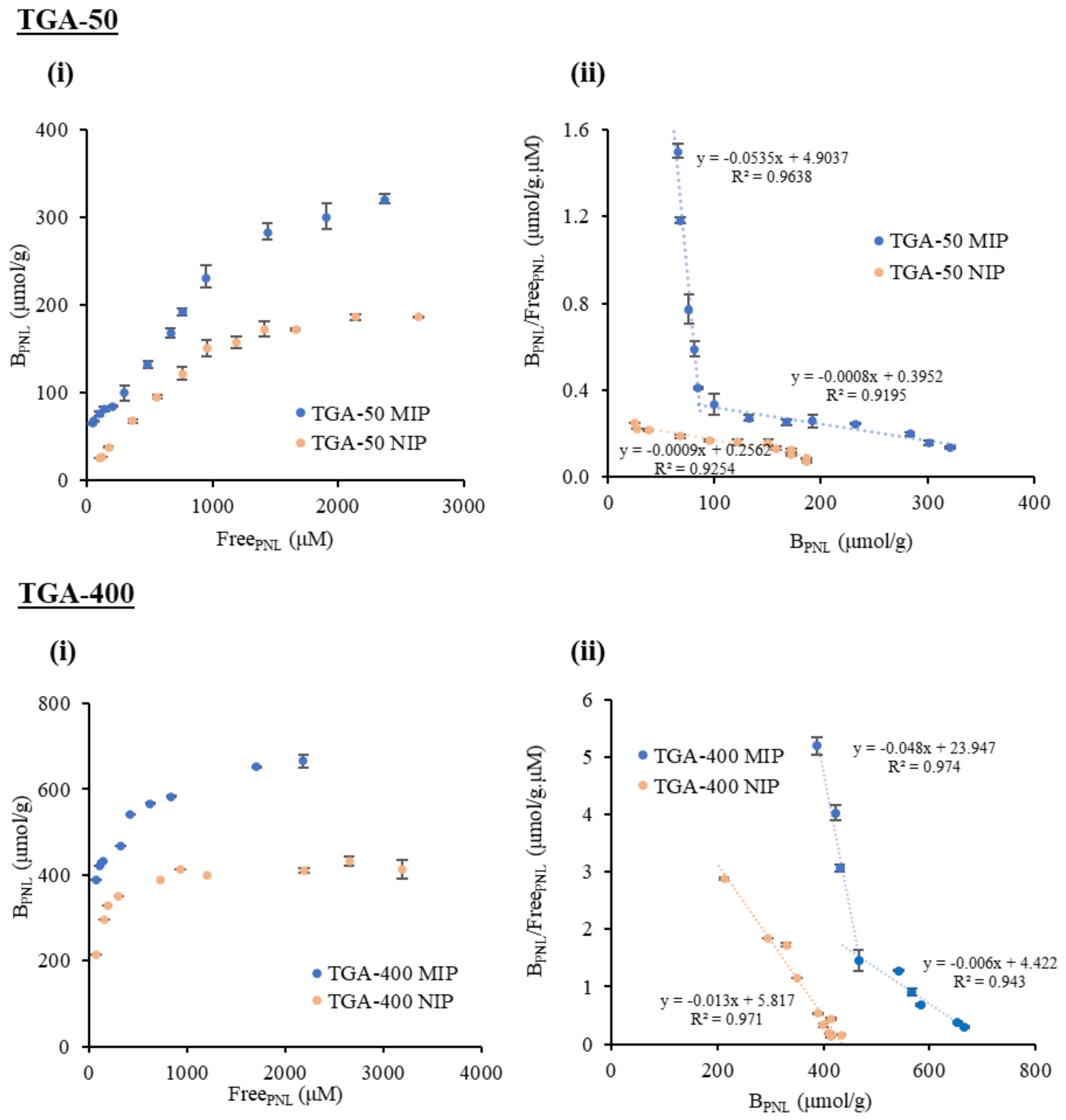

Figure S2. Langmuir binding isotherms **(i)** and Scatchard plots **(ii)** for TGA-50 and TGA-400 MIPs and NIPs. The Scatchard plots for MIPs show two gradients representing high affinity (blue line with steeper gradient) and low affinity binding sites. All binding tests were conducted in triplicates for each of 4 batches of polymers.

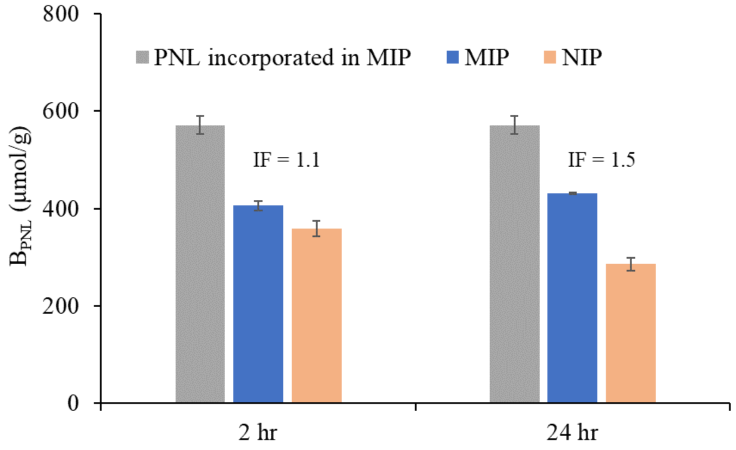

Figure S3. Time-binding tests for the TGA/MAA MIP and NIP. 2.0 mg polymers were incubated at 2 and 4 hours with 1.0 mL of 2 mM PNL solution. Rebinding was quantified by q-HNMR. All binding tests were conducted in single run for each of 4 batches of polymers.

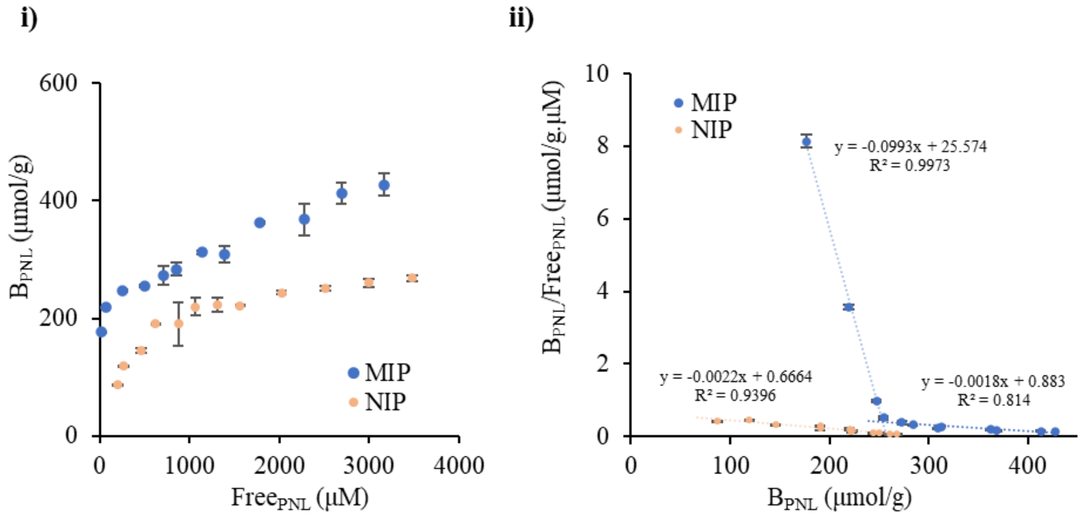

Figure S4. Langmuir binding isotherms **(i)** and Scatchard plots **(ii)** for TGA/MAA MIP and NIP. The Scatchard plot for MIP show two gradients representing high affinity and low affinity binding sites. All binding tests were conducted in duplicates for each of 4 batches of polymers.

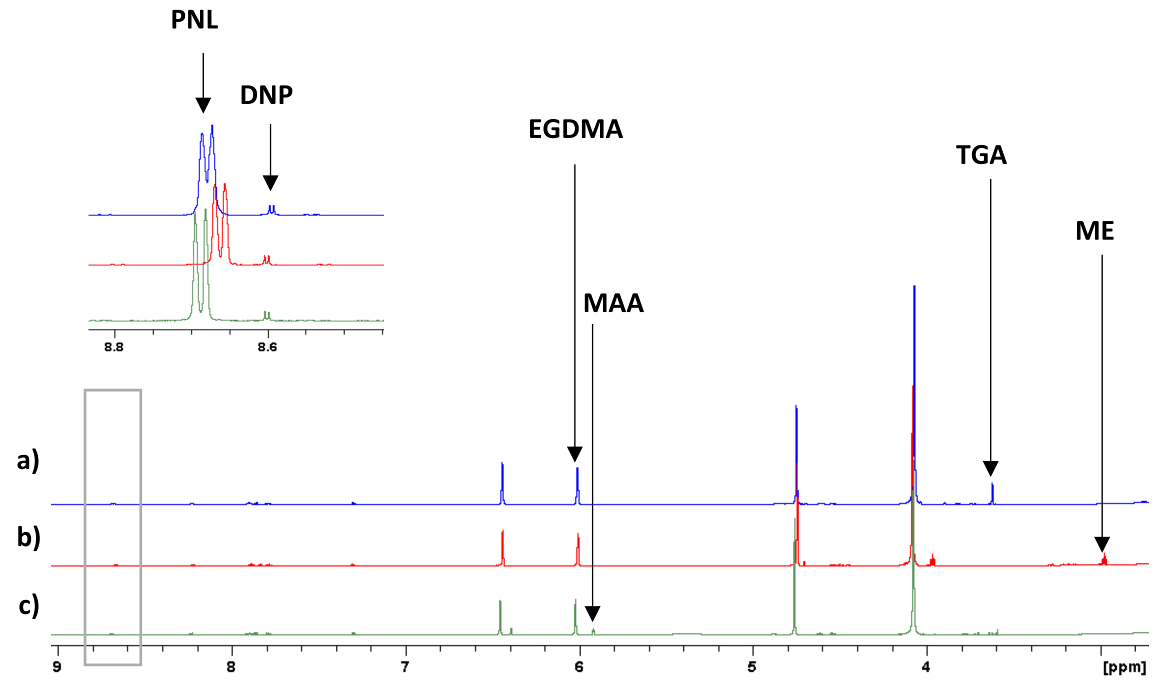

Figure S5. ^{1}H-NMR spectra of: **a)** TGA-400 MIP, **b)** ME-400 MIP and **c)** TGA/MAA MIP pre-polymerisation solutions showing peaks used for quantitative analysis: template **PNL** - Ar-**H** signal at 8.70 ppm, internal standard **DNP** - Ar-**H** signal at 8.65 ppm, cross-linker **EGDMA** -CR=C**H**$_2$ signal at 6.02 ppm, functional monomer **MAA -** CR=C**H**$_2$ signal at 5.90 ppm, chain transfer agent **TGA** - HS-C**H**$_2$- signal 3.60. Note: Signal at 3.00 ppm is for -C**H**$_2$-OH of another chain transfer agent mercaptoethanol **ME** and not included in this paper.

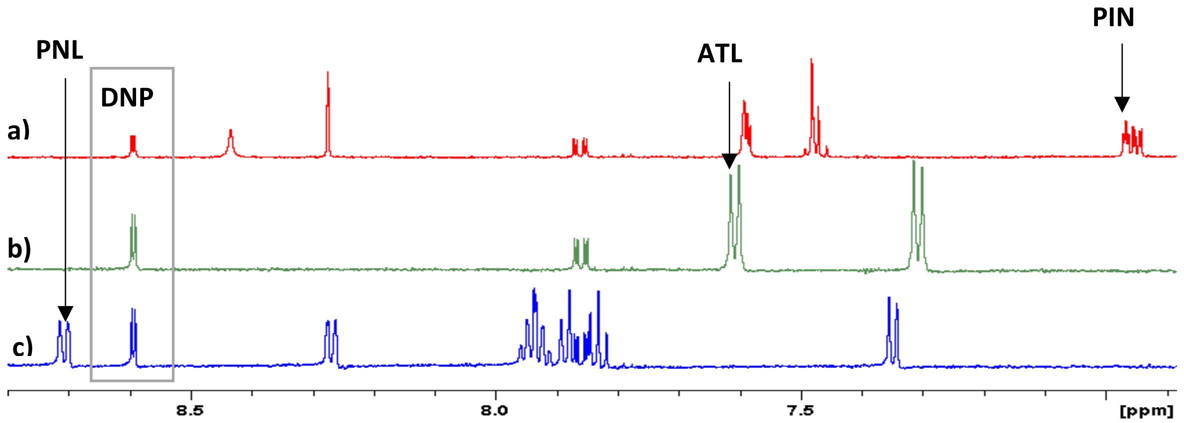

Figure S6.^{1}H-NMR spectra of **a)** 2 mM pindolol (**PIN**), **b)** 2 mM atenolol (**ATL**) and **c)** 2 mM propranolol (**PNL)** with 10 mM 2,4-dinitrophenol (**DNP**) in DMSO-d_6 as external standard in a 5-mm probe coaxial insert. Peaks used for q-HNMR are shown as follows: **PNL** - Ar-**H** signal at 8.70 ppm, **DNP** - Ar-**H** signal at 8.60 ppm, **ATL** - Ar-**H** signal at 7.60 ppm and **PIN** - Ar-**H** signal at 6.95 ppm.
